# Motivational decline and proactive response under thermal environmental stress are related to emotion- and problem-focused coping, respectively: Questionnaire construction and fMRI study

**DOI:** 10.3389/fnbeh.2023.1143450

**Published:** 2023-04-12

**Authors:** Kelssy Hitomi dos Santos Kawata, Kanan Hirano, Yumi Hamamoto, Hajime Oi, Akitake Kanno, Ryuta Kawashima, Motoaki Sugiura

**Affiliations:** ^1^Institute of Development, Aging and Cancer, Tohoku University, Sendai, Japan; ^2^Graduate School of Medicine, The University of Tokyo, Tokyo, Japan; ^3^Graduate School of Interdisciplinary Information Studies, The University of Tokyo, Tokyo, Japan; ^4^Climate Control and Cooling System Engineering Group, Nissan Motor Co., Ltd., Atsugi, Japan; ^5^Graduate School of Medicine, Tohoku University, Sendai, Japan; ^6^Graduate School of Engineering, Tohoku University, Sendai, Japan; ^7^International Research Institute of Disaster Science, Tohoku University, Sendai, Japan

**Keywords:** coping, stress, thermal, fMRI, discomfort, emotion regulation, thermal stress, psychological responses

## Abstract

Despite the diversity of human behavioral and psychological responses to environmental thermal stress, the major dimensions of these responses have not been formulated. Accordingly, the relevance of these responses to a framework of coping with stress (i.e., emotion- and problem-focused) and the neural correlates are unexplored. In this study, we first developed a multidimensional inventory for such responses using social surveys and a factor analysis, and then examined the neural correlates of each dimension using a functional magnetic resonance imaging; we manipulated the ambient temperature between uncomfortably hot and cold, and the correlations between the inventory factor scores and discomfort-related neural responses were examined. We identified three factors to construct the inventory: motivational decline, proactive response, and an active behavior, which appeared to reflect inefficient emotion-focused coping, efficient problem-focused coping, and positive appreciation of extreme environmental temperatures, respectively, under environmental thermal stress. Motivational decline score was positively associated with common neural response to thermal stress in the frontal and temporoparietal regions, implicated in emotion regulation, while proactive response score negatively with the neural responses related to subjective discomfort in the medial and lateral parietal cortices, implicated in problem-solving. We thus demonstrated that two of three major dimensions of individual variation in response to and coping with environmental thermal stress conform to an influential two-dimensional framework of stress coping. The current three-dimensional model may expand the frontiers of meteorological human science in both basic and application domains.

## Graphical Abstract

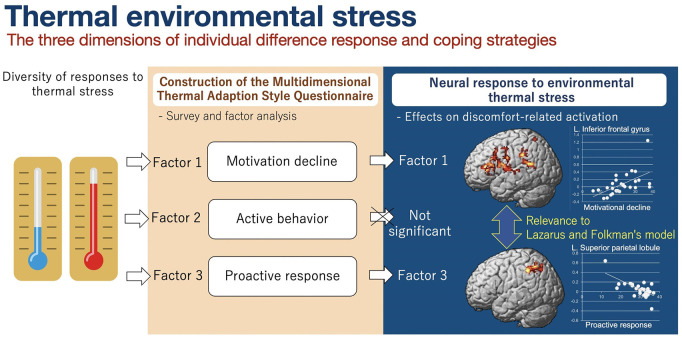

## 1. Introduction

Humans live in diverse thermal environments on earth (Jendritzky and Tinz, 2009). Rarely are temperatures within the range of human comfort, and all people cope with thermal environmental stress through technical, behavioral, or psychological methods (Gao et al., 2018; Coudevylle et al., 2019). Interestingly, a great deal of individual variation exists in how people respond and prefer to cope with thermal stress (Foster et al., 2020). That is, the individual difference in such responses or coping strategies may have multiple dimensions: for example, the difference can have an aspect of vulnerability or tolerance to temperature changes (Shi et al., 2016), that of the level of preparedness to the change (Tochihara et al., 2022), and that of the degree how one enjoys the change (Nikolopoulou et al., 2001).

Understanding such multiple dimensions in the diversity of responses and coping strategies to thermal environmental stress and their psycho-behavioral or neural underpinning may provide more effective ways to cope with thermal environmental stress (Salomons et al., 2007; Coudevylle et al., 2019). This is an issue of wellbeing (Tham, 2004; Li et al., 2021), due to the decreased physical condition (Coudevylle et al., 2019) and cognitive performance (Laurent et al., 2018), as well as increased mortality (Huynen et al., 2001), related to thermal environmental stress. In addition, this is significant in terms of the growing importance of reducing energy for air conditioning and sustainability (Fukusaka and Matsubara, 2014). On the other hand, it may also contribute to an anthropological understanding of why humans can adapt to a wide range of thermal environments and have become distributed more ubiquitously across the globe than other animal species (Hanna and Brown, 1983; Tochihara et al., 2022).

An influential conceptual framework for multiple dimensions in stress-coping in general is the Lazarus and Folkman’s model (Folkman and Lazarus, 1980, 1985), which categorizes stress coping strategies into emotion-focused coping and problem-focused coping strategies. Emotion-focused coping strategies (also referred to as antecedent-focused) involve regulating the emotional reaction that the stressor elicited; these occur in two forms of adaptive emotion regulation strategies, such as restructuring and reinterpreting a negative situation (reappraisal), or accepting negative situations more easily (acceptance) (Wolters et al., 2010; Aldao and Nolen-Hoeksema, 2012; Shin et al., 2014). Emotion-regulation failure of this adaptive strategy is thought to be responsible for some psychopathology, such as mood-related disorders (e.g., depression and anxiety), externalizing disorders (e.g., substance use and eating disorders) (Aldao et al., 2010), and anti-social attitudes (i.e., as a consequence of regulatory failure) (Koenigsberg et al., 2009). In contrast, the adaptive emotion regulatory strategy related to the problem-focused coping strategy (also referred to as proactive coping) (Aldao et al., 2010) attempts to focus directly on the problem and to solve the existing problem (i.e., stress) (Diponegoro et al., 2020). Individuals plan the next step or incorporate information to mobilize actions to change the situation (Lazarus, 1991; Van Zomeren et al., 2010).

These two dimensions of stress-coping strategies may be relevant to neural processes at different stages of emotion processing. A successful emotion-focused coping strategy (i.e., reappraisal or acceptance) is assumed to tap into the early stage detection process, or the first-level valuation of emotion in an influential process model of emotion (Gross, 1998b) as well as the acceptance of mindfulness (Ho et al., 2015). Individuals who efficiently use this type of strategy prevent this first-level valuation developing into second-level valuation, which encompasses emotional responses in various domains (i.e., consciousness, physiology, and behavior) linked to emotional stress (Öner, 2018). Neuroimaging studies have explored neural correlates of such a capacity using correlational analysis and trait measures of emotion-regulation capacity scores. They compared groups with different levels of neural activation capacity during manipulation of emotional stress caused by various stressors (Salomons et al., 2007; Preis et al., 2015; Kober et al., 2019; Messina et al., 2021), such as unpleasant scenes, thermal pain, negative autobiographical memories, one-sentence stressful scenarios, worry statements, and negative self-belief statements. Most of these studies demonstrated that adaptive individuals present with less activation of the brain regions involved in the emotional control response and modulation of emotion-generating processes, such as limbic (e.g., amygdala, insula) and semantic (e.g., superior temporal gyrus; STG) related brain structures, respectively, under potential emotional stress (Preis et al., 2015; Kober et al., 2019; Messina et al., 2021). However, the role of the executive function system remains controversial (e.g., dorsolateral prefrontal cortex; DLPFC, ventrolateral prefrontal cortex; VLPFC), which is used with various emotion regulation strategies (Salomons et al., 2007; Kober et al., 2019; Messina et al., 2021). Some studies have suggested that adaptive individuals show greater activation, reflecting the appropriate use of these strategies (Salomons et al., 2007) while others have suggested the opposite (Kober et al., 2019; Messina et al., 2021), arguing that involvement reflects the emotional response itself, inefficient use of the strategy, or dependence on the type of instruction (i.e., natural reaction or focus on emotion).

On the other hand, it is theoretically expected that the capacity to problem-focus cope is reflected in the late stage of emotion processing, that is, when one is aware of the emotional stress and the problem behind the stress is worth solving. Neuroimaging studies have suggested the involvement of lateral and medial parietal cortices in such creative problem-solving (Bartley et al., 2018) and future thinking (Stawarczyk and D’Argembeau, 2015; Schacter et al., 2017), but the view remains mixed regarding how spontaneous engagement of such problem-solving systems is related to individual problem-solving capacity. Some studies have suggested more engagement of the system in higher-capacity individuals. For example, high problem-solving performance is associated with greater activation of the medial parietal region during the performance of an unrelated task before explicit problem-solving (Kageyama et al., 2019) and young adults rich in social experience reveal high activity in the lateral parietal cortex during judgment of the appropriateness of the use of honorific expressions (Cui et al., 2022). Other studies have suggested the opposite; high problem-solving performance is associated with minimal activation of the lateral parietal during the management of unexpected trouble in a realistic plant-control simulator (Miura et al., 2020) and high decision-making speed with low temporoparietal activation during realistic social-problem solving (recommending clothing in a difficult social context) (Oba et al., 2020).

Regarding the thermal environmental stress, however, dimensions in the responses and coping strategies has been yet to be formulated and accordingly their psycho-behavioral or neural underpinning are yet to be explored. Previous relevant meteorological studies relied on a unidimensional vulnerability/tolerance construct to investigated individual factors (e.g., age, sex, emotional state) that affect thermal environmental stress and the cognitive processes (Wang et al., 2018; Zhang et al., 2019). Cognitive neuroscience of environmental thermal perception remains its infancy (Oi et al., 2017) and individual difference in the responses or coping strategies has not been explored.

In this study, we aimed at formulating major dimensions of the individual difference in responses or coping strategies to thermal environmental stress and understand the neural correlates of each dimension. To this end, we first created a multidimensional inventory (Multidimensional Thermal Adaption Style Questionnaire; MTASQ) by collecting a wide range of people’s daily responses or coping strategies to thermal environmental stress and then identifying their major dimensions using a factor analysis (Comrey, 1988). We also explored the correlation between each MTASQ dimension score and various demographic, physiological, and psycho-behavioral variables to validate and characterize each dimension; we were interested in if the identified dimensions conform to Lazarus and Folkman’s model and how they are related to adaptability based on anthropological interest. For psycho-behavioral variables, we used two questionnaires: one known as the eight factors of the “Power to Live,” which was advantageous for survival in various disaster contexts (Sugiura et al., 2015), and another known as the Big Five General Personality Traits (Gosling et al., 2003; Oshio et al., 2012). The former was included based primarily on anthropological interest because the eight factors are relevant to different survival-relevant processes in various physical (Sugiura et al., 2019; Sato et al., 2021), social (Sugiura et al., 2020, 2021), and developmental (Matsuzaki et al., 2022; Sugiura, 2022) contexts. The latter, together with demographic and physiological variables, were used to compare their correlation pattern between the MTASQ dimensions and two types of stress-coping strategy (i.e., emotion- and problem-focused) in Lazarus and Folkman’s model, for which knowledge on such correlation patters are available (Chen et al., 2018; Agbaria and Mokh, 2022). We expected that some of the MTASQ dimensions would be conformal to emotion-focused or problem-focused strategy.

We then investigated the neural correlates of each MTASQ dimension during uncomfortable thermal environmental stress using fMRI. We manipulated the ambient temperature in the gantry of the scanner between uncomfortably hot and cold ranges and obtained the time-series data of brain activation and rating of subjective level of discomfort. For the analyses, we adopted two neural-activation models to extract different types of thermal discomfort-related neural response relevant to different stages of emotion processing (Gross, 1998a). The first model was created to capture the detection process of thermal discomfort at an early stage, which was the first level of valuation before the second-level valuation or the emotional responses. Assuming that the process was unaffected by individual emotion-focused coping and largely the same across participants, the model was created by averaging the time-series data of the discomfort rating across all the participants (average model). The second model was created to capture responses related to subjective perception of thermal discomfort associated with a late stage, second-level valuation, or an emotional response. The model was individually tailored using a subjective rating for each participant (individual model). We expected that the scores of the emotion-focused MTASQ dimension would be correlated with activation in emotion-regulation-related brain regions in the average model, and that those of the problem-focused MTASQ dimension would be correlated with the parietal cortices in the individual model.

## 2. Materials and methods

The research was composed two components. First, we created a multidimensional inventory (i.e., MTASQ) for individual difference in the responses or coping strategies to thermal environmental stress, and explored the correlation between each dimension score and various demographic, physiological, and psycho-behavioral variables to validate and characterize each dimension (2.1). Second, we investigated the neural correlates of each MTASQ dimension during uncomfortable thermal environmental stress using fMRI (2.2).

### 2.1. Construction of the MTASQ

A qualitative survey was initially performed to prepare the candidate inventory items, and then a quantitative survey was conducted to perform a factor analysis of these candidate items. The latter survey also included variables to assess the criterion-related validity of the identified factors. The survey protocols were reviewed and approved by the Ethics Committee for Surveys and Experiments at the International Research Institute of Disaster Sciences, Tohoku University (2016-013).

#### 2.1.1. Participants

The two surveys were conducted by a crowd-sourcing company (Cross Marketing Inc., Tokyo, Japan). The respondents were recruited from the company’s registered panel in Japan and participated in exchange for an online voucher/shopping points. The participants were stratified into five age classes (20s, 30s, 40s, 50s, and 60s) and two sexes (male or female) for each survey. We intended to have 20 and 120 respondents for each class after data pre-screening (excluding straight-line responders) in the qualitative and quantitative surveys, respectively. As a result, we obtained 20–22 (*n* = 203 in total) and 114–145 (*n* = 1,327 in total), respectively. See Supplementary Table 1 for the detailed statistics of the responders. The recruitment of the responders for the two surveys was conducted separately, but the potential overlap of the responders between the two surveys could not be assessed due to the anonymous nature of the surveys.

#### 2.1.2. Qualitative survey

Responders were asked to provide ten first-person-perspective descriptions for “what they think, do, or want to do” when “the temperature gets hotter” and when “the temperature gets colder”. They were also asked to provide five third-person-perspective descriptions of a person who is tolerant of heat, a person who is sensitive to heat, a person who is sensitive to cold, and a person who is tolerant of cold, regarding their “characteristics and what they are likely to think or do”.

All of the descriptions were pooled and sorted by their meaning and context. Through extensive discussions with the authors, the descriptions were summarized and formatted into phrases for a self-applicability rating, resulting in 70 candidate items for the hot environment and 70 for the cold environment. There were many common or similar items between the hot and cold environments.

#### 2.1.3. Quantitative survey

Respondents were presented with 70 candidate questionnaire items for the hot environment (“When it’s hot,”) and 70 for the cold (“When it’s cold,”) and were asked for a self-applicability rating on a 7-point scale (1: *not at all applicable*; 7: *very much applicable*).

The participants responded to another 54 items to generate 22 variables to assess the criterion-related validity of the factors. Demographic variables included sex and age. Physiological or psychological variables related to thermal perception included body mass index (BMI = weight/height^2^), subjective levels of tolerance/intolerance to hot/cold environment (2 × 2 = 4 variables; “I think I am a hot/cold tolerant/sensitive person,”) on a 7-point scale (1: *not at all applicable*; 7: *very much applicable*), and the tolerable range of ambient temperatures (in °C). The “Power to Live” scale was comprised of 34 items on a 6-point scale (0: *not at all*; 5: *very much*), with eight factors: leadership, problem-solving, altruism, stubbornness, etiquette, emotion regulation, self-transcendence, and active wellbeing. The internal consistency and concurrent validity of the questionnaire have been demonstrated (Sugiura et al., 2015; Ishibashi et al., 2019). We used the Japanese version of the Ten-Item Personality Inventory for the Big Five Personality Scale (Gosling et al., 2003; Oshio et al., 2012), which includes one positive item and one reverse-scored item on a 6-point scale (0: *not at all*; 5: *very much*) for each of the five factors, including extraversion, agreeableness, conscientiousness, neuroticism, and openness. We adopted this very short version of the Big Five Inventory to minimize fatigue or frustration, which could decrease the rate and quality of the responses. The validity of this short version of the Big Five Inventory has been established in terms of convergent and discriminant validity, coverage of sub-dimensions, test–retest reliability, and patterns of external correlates. The sum of the scores for each factor or dimension (the scores of reverse items were reverse coded) was converted to a ratio against the maximum score.

#### 2.1.4. Analysis

A factor analysis was performed on the ratings of the 140 candidate items using a maximum likelihood method to determine the major factors associated with the individual differences in the responses to environmental thermal stress. The number of factors to include was determined using a scree plot. The Promax rotation method was applied.

We expected to identify factors related to the responses to general environmental thermal stress rather than those specific to a hot or cold environment. As a result, similar psychological or behavioral responses to hot and cold environments were largely clustered in the factor analysis (see section “3. Results”). Therefore, we decided to include similar hotness-related and coldness-related responses as a pair and select the three pairs with the highest average factor loadings. This resulted in a list of three typical responses to the uncomfortable thermal environment for each factor without specific responses to the hot or cold environment. By limiting the pairs to three, the questionnaire was kept concise enough to be easily used. The internal reliability of each factor (i.e., six items) was evaluated using Cronbach’s α.

Criterion-related validity of each factor was assessed using associations between the factor score (i.e., the total score of six items) and the demographic, physiological, and psycho-behavioral variables. Because a hypothesis of normal distribution was rejected (*p* < 0.05, Shapiro-Wilk test) in all the three factors, non-parametric tests were used. The effect of sex was assessed using a Mann-Whitney *U*-test (Mann and Whitney, 1947) and the effects of the other variables were tested using Spearman’s correlation analysis (Spearman, 1961). We also examined the cross-correlation among MTASQ factors. We report *p*-statistics at an uncorrected *p* < 0.05 and a Bonferroni’s corrected *p* (*total number of tested associations across all variables and factors) <0.05 level. However, we selected the significant associations based on the effect-size criteria given too much statistical sensitivity due to a large sample size (Cohen, 1992): large, medium, and small size effects for Vargha and Delaney’s A >0.71, 0.64, and 0.56, or <0.29, 0.34, and 0.44 (Vargha and Delaney, 2000), and Spearman’s ρ > 0.5, 0.3, and 0.1 (Ellis, 2010), respectively. The analyses were performed with free R (version 4.2.2) statistical software by using the *stats*, *psych*, and *effsize* packages.

### 2.2. fMRI

#### 2.2.1. Participants

Forty-six healthy right-handed adults (mean age = 21.2 years, *SD* = 1.6, age range = 18.0–25.0 years, 15 females) were recruited from Tohoku University, Sendai, Japan. The experiment was performed during July–September. Written informed consent was obtained from each participant. The experiment was conducted following the Declaration of Helsinki, and all procedures were approved by the Institutional Review Board of the Tohoku University, Graduate School of Medicine, and the Nissan Motor Ethics Committee.

#### 2.2.2. Experimental equipment and procedures

The participants adjusted their clothes until they felt comfortable in the ambient temperature of the scanner room (approximately 22°C). Strict control over clothing was avoided to match more closely the study environment with that of the participant’s daily life experience. Each participant lay on the MRI scanner bed with a temperature transducer attached to the scanner bed, close to the MRI head coil, and the participant’s head was fixed to the head coil using a band and foam blocks. The participants were covered with a large plastic canopy fit to the size of the MRI gantry, and hot or cold air produced using an air conditioner located outside the scanner room was blown into the canopy through a duct (Oi et al., 2017). During the fMRI measurement, 10 min heating and 10 min cooling phases were alternated twice; the session began with the heating phase for half of the participants and with the cooling phase for the other half. All participants were asked to rate independently their subjective levels of thermal sensation and discomfort/comfort every 30 s. The temperature change was intended to range between uncomfortably hot or cold with a comfortable range in between (mean = 23.1°C, mean range = 19.3–26.2°C, *SD* = 1.3), which would cause the frequency of the time-series changes in thermal uncomfortable ratings to be double those of the thermal sensation ratings; thus, the two ratings were independent. After the fMRI scanning and tasks were completed, each participant was removed from the scanner and completed the MTASQ rating.

#### 2.2.3. fMRI task

The ratings of subjective levels of thermal sensation and discomfort/comfort were alternately instructed separated by 15 s interval. A 4-button MRI-compatible pad was held in each hand. One pad was used for the four-point scale to indicate thermal sensation (1: *cold*, 2: *cool*, 3: *warm*, and 4: *hot*), while the other pad was used for the four-point scale to indicate thermal comfort (1: *discomfort*, 2: *slight discomfort*, 3: *slightly comfortable*, and 4: *comfortable*). The button arrangements were counterbalanced across all participants. The task instructions were presented *via* MRI-compatible goggles. In each trial, an instruction for the button assignment was visually presented for 5 s, during which the participant was required to respond, followed by presentation of a fixation cross for 10 s. In total 80 sensation and 80 comfort trials were performed over 40 min. The task was controlled by PsychoPy v1.83.03, which presented the stimuli and recorded the participants’ ratings for later analysis.

#### 2.2.4. fMRI data acquisition and pre-processing

All fMRI data were acquired with a 3T Philips Achieva scanner (Philips Healthcare, Best, Netherlands) using an echo-planar sequence sensitive to the blood oxygenation level-dependent contrast with the following parameters: 64 × 64 matrix, repetition time (TR) = 2,500 ms, TE = 30 ms, flip angle = 85°, FOV = 192 mm^2^, 39 slices, slice thickness = 3.0 mm and gap = 0.5 mm. A total of 960 volumes were acquired during the session.

The following preprocessing procedures were performed using Statistical Parametric Mapping (SPM12) software (Wellcome Department of Imaging Neuroscience, London, UK) and MATLAB (Mathworks, Natick, MA, USA): acquisition timing across slices was adjusted and head motion was corrected and normalized to the Montreal Neurological Institute (MNI) reference space using the EPI template and smoothed with an isotropic Gaussian kernel with 5 mm full-width at half-maximum. The choice of the smoothing-kernel size was based on our previous study (Oi et al., 2017).

#### 2.2.5. Behavioral data analyses

We conducted a Spearman’s correlation analysis between the MTASQ scores of the participants and the thermal sensation and comfort ratings during the fMRI experiment, expecting a relationship between a high emotion-focused coping tendency and sensitivity to discomfort (Amen, 2008; Schoenmakers et al., 2015). The average thermal sensation and comfort rating scores were used. Analyses were performed using SPSS version 25 for Windows and a *p* < 0.05 and 0.10 were considered significant and tendency of correlation, respectively.

#### 2.2.6. fMRI data analyses

A conventional two-level fMRI analysis was adopted using SPM12 (Friston et al., 1990, 1991). The heat (i.e., based on sensation rating) and discomfort (i.e., based on reverse-coded comfort rating) perceptions were modeled as a general linear model (GLM) for the first-level analysis. The individual model and average model were modeled as separate GLMs. The individual model used the raw rating score of each participant, as in our previous study (Oi et al., 2017). The average model, which was newly adopted in this study, used an average rating across all participants at each time point. The ratings were linearly interpolated at each scan time (i.e., TR = 2.5 s). The convolution of the hemodynamic response function was not adopted considering the significant delay in the rating from the actual thermal sensation. Six estimated head motion parameters were included in the GLMs as confounding factors. High-pass filtering with a frequency cut-off at cycle/1,200 s (i.e., twice the cycle of the change in the comfort rating) was applied to reduce low-frequency noise in the brain activity.

As a second-level analysis, two approaches were adopted to investigate effects of the MTASQ factors on discomfort-related neural activation in the regression analysis. For the first approach, we initially identified discomfort-related activation using a one-sample *t*-test, and then examined the effect of the MTASQ factors at the peak voxels of the identified activation clusters using a liberal statistical threshold for the region-of-interest (ROI) regression analysis. This approach assumed a modest level of individual difference. For the second approach, voxel-wise simple regression analyses using the MTASQ scores were directly performed; this approach assumed robust individual differences in activation, which may have prevented detection of discomfort-related activation using an across-participant one-sample *t*-test. For each approach, discomfort-related activation was estimated using the individual model and the average model separately, and analyzed for distinct MTASQ factors independently; gender was included as a covariate. As a *post hoc* analysis for the first approach, a one-sample *t*-test on the average model at the peak voxels was identified using the one-sample *t*-test on the individual model, and vice versa, were also applied. For the second approach, the same MTASQ regression on the average model at the peak voxels identified using the same MTASQ regression on the individual model, and vice versa, were also applied. The voxel-wise statistics used an uncorrected *p* < 0.005 for the cluster-forming threshold, which was thresholded at a family wise error-corrected *p* < 0.05 for cluster extent, following our previous study (Oi et al., 2017). The statistical threshold for the ROI analyses was set to an uncorrected *p* < 0.05. The identified brain structures were anatomically labeled using the SPM Anatomy toolbox (Eickhoff et al., 2005).

## 3. Results

### 3.1. MTASQ

#### 3.1.1. Factor analysis

We accepted a three-factor solution based on the scree plot, which showed an abrupt drop in loading between the third and fourth factors and a subsequent gradual decline (30.9, 9.95, 6.24, 3.72, 3.16, 2.66, 2.49…). The results of the factor analysis after selecting six items (i.e., three pairs) for each factor are given in Table 1. The first factor was composed of loss of smile, decreased talk, and negative thoughts in an uncomfortable thermal environment, which we interpreted commonly to reflect “Motivational decline.” The second factor was composed of “Active behaviors” including an attempt to sweat (sauna and heavy clothing), physical exercise, and going out. The third factor was composed of precaution actions aimed at preventing adverse effects of the uncomfortable thermal environment or related weather, such as attention to the weather forecast, drinks, food, and skin, which we labeled the “Proactive response.” Each item had a loading of ≥0.404 on the relevant factor and ≤0.142 on the other factors. Each factor had good internal consistency and reliability with Cronbach’s α ≥ 0.728.

**TABLE 1:** Results of factor analysis.

Environment	Items	F1	F2	F3
**F1 motivational decline (α = 0.869)**				
Hot	I don’t smile as much	**0**.**687**	-0.015	-0.028
Cold	I don’t smile as much	**0**.**867**	0.024	-0.049
Hot	I don’t talk a lot	**0**.**676**	-0.061	-0.017
Cold	I don’t talk a lot	**0**.**836**	0.015	-0.035
Hot	I have negative thoughts	**0**.**578**	-0.034	0.136
Cold	I have negative thoughts	**0**.**676**	0.004	0.113
**F2 active behavior (α = 0.794)**				
Hot	I try to sweat (sauna, heavy clothing, etc.)	0.003	**0**.**706**	-0.039
Cold	I try to sweat (sauna, heavy clothing, etc.)	0.060	**0**.**676**	0.084
Hot	I exercise (sports, training, etc.)	-0.111	**0**.**735**	0.082
Cold	I do winter sports (skiing, snowboarding, etc.)	0.086	**0**.**593**	-0.142
Hot	I go camping and barbecuing	0.022	**0**.**525**	-0.055
Cold	I actively go out	0.025	**0**.**487**	0.090
**F3 proactive response (α = 0.728)**				
Hot	I pay attention to the weather forecast	-0.003	0.059	**0**.**644**
Cold	I pay attention to the weather forecast	0.059	0.039	**0**.**668**
Hot	I hydrate frequently	-0.128	0.024	**0**.**464**
Cold	I take warm drinks and food	-0.110	-0.074	**0**.**612**
Hot	I take care not to get sunburned	0.053	-0.012	**0**.**404**
Cold	I worry about dryness	0.012	0.003	**0**.**589**

The results of the final factor analysis after selecting three pairs of the same or similar psychological or behavioral responses to the hot and cold environment. The items in a pair (i.e., for the hot and cold environments) and their factor loadings (in bold indicates >0.5) are given for each factor; the pairs are listed in the order of the average factor loading. Hot and cold for the environment indicate that the question is asked about hot (“When it’s hot,”) and cold (“When it’s cold,”) environments, respectively. α: Cronbach’s α.

#### 3.1.2. Criterion-related validity

Table 2 shows the association between the factor scores and the demographic, physiological, and psycho-behavioral variables, and the scores for the other MTASQ factors. Several associations with the Motivational decline score (F1) had a small effect size, including a lower age, lower subjective tolerance for a hot environment, higher subjective intolerance for a hot or cold environment, higher tolerable low temperature, less problem solving, etiquette, emotion regulation, extraversion, agreeableness, conscientiousness, and higher neuroticism. Associations with a medium effect size for the Active behavior (F2) score were identified for higher leadership and active wellbeing. Additionally, associations with a small effect size were observed for the male sex, with higher subjective tolerance and lower subjective intolerance to a hot environment, greater problem-solving, altruism, stubbornness, emotion regulation, self-transcendence, extraversion, conscientiousness, openness, and lower neuroticism. Associations with a medium effect size for the Proactive response (F3) score were identified for the female sex and greater etiquette. In addition, associations with a small effect size were observed for smaller BMI, higher subjective intolerance to a hot or cold environment, greater problem-solving, altruism, stubbornness, emotion regulation, self-transcendence, active wellbeing, and agreeableness. Among the three MTASQ factors, the Motivational decline (F1) score was correlated positively with that of Active behavior (F2) with small effect size; no significant correlation was detected between the Proactive response (F3) and other two factors.

**TABLE 2:** Associations between the scores on the Multidimensional Thermal Adaptation Style Questionnaire and the variables.

		F1 motivational decline	F2 active behavior	F3 proactive response
Mean ± standard deviation				
Sex	Male	19.0 ± 7.5	17.7 ± 6.8	26.9 ± 5.9
	Female	19.4 ± 6.8	15.6 ± 6.5	31.8 ± 5.8
	*U*	222.246	179.069**	320.115**
	*A*	0.507	*0.408*	**0.730**
Spearman’s ρ				
Age		*−0.112* **	−0.029	0.018
BMI		−0.064*	0.021	*−0.169* **
Subjective tolerance	Hot	*−0.100* **	*0.209* **	−0.053
	Cold	−0.083*	0.074*	0.014
Subjective intolerance	Hot	*0.197* **	*−0.120* **	*0.176* **
	Cold	*0.181* **	−0.005	*0.114* **
Tolerable temperature	Max.	−0.098**	0.090*	−0.052
	Min.	*0.114* **	−0.034	0.038
Power to live	Leadership	−0.035	**0.430** **	0.003
	Problem solving	*−0.129* **	*0.145* **	*0.270* **
	Altruism	0.039	*0.279* **	*0.150* **
	Stubbornness	0.012	*0.121* **	*0.189* **
	Etiquette	*−0.214* **	−0.015	**0.378** **
	Emotion regulation	*−0.147* **	*0.204* **	*0.149* **
	Self-transcendence	−0.052	*0.189* **	*0.236* **
	Active wellbeing	−0.032	**0.358** **	*0.180* **
Big five	Extraversion	*−0.201* **	*0.285* **	−0.032
	Agreeableness	*−0.239* **	−0.042	*0.199* **
	Conscientiousness	*−0.198* **	*0.104* **	0.023
	Neuroticism	*0.278* **	*−0.159* **	0.097**
	Openness	−0.065*	*0.268* **	−0.059*
MTASQ	F1 Motivational decline		*0.258* **	0.083*
	F2 Active behavior			−0.068*

The effect of sex was assessed using a Mann-Whitney *U*-test and the other variables were tested using Spearman’s correlation analysis. We reported *p*-statistics at uncorrected *p* < 0.05 (*) and Bonferroni’s corrected *p* < 0.05 (**; #tests = 69) levels. Significant associations were identified based on the effect-size criteria due to the high sensitivity caused by a large sample size (Cohen, 1992): medium (in bold) and small (in Italic) effect sizes for Vargha and Delaney’s A > 0.64 or < 0.34 and > 0.56 or < 0.44 (Vargha and Delaney, 2000), and Spearman’s ρ > 0.3 and 0.1 (Ellis, 2010), respectively.

### 3.2. fMRI

Eighteen participants were excluded from the fMRI analysis: two participants because of a technical problem associated with the capture of the behavioral response during the task, three participants showed excessive head motion (>7 mm) within the scanner; 11 participants reported insufficient variation in the level of comfort pressing discomfort or comfort buttons less than 3.75%, and the other two participants had problems with their normalized fMRI data during preprocessing, so these data were excluded from the statistical analysis. Thus, after the exclusions, the data from 28 participants (mean age = 21.2 years, *SD* = 1.3, age range = 19.0–24.0 years, 7 females) were included in the final analysis for the discomfort/comfort thermal perception investigation.

#### 3.2.1. Behavioral data

**FIGURE 1:**
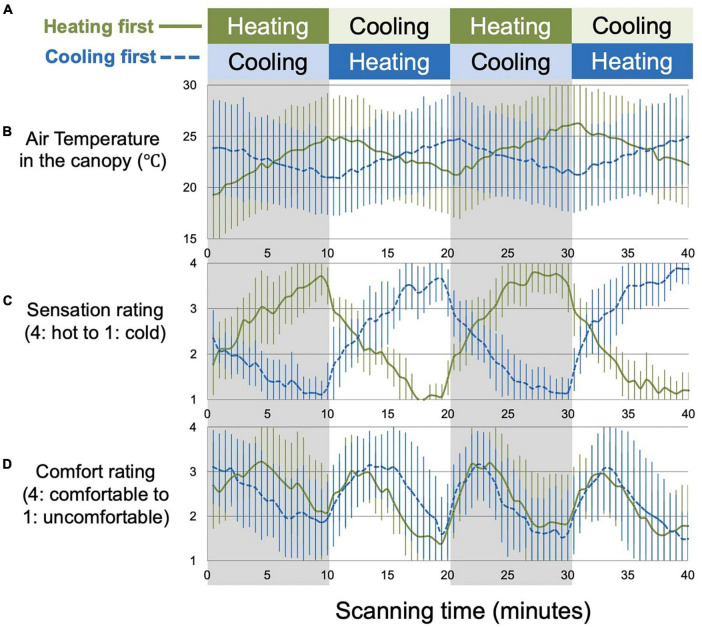
Thermal manipulation and perceptual measures. Each session consisted of two alternations of 10 min heating and 10 min cooling phases; the session began with the heating phase (heating first) for 14 participants and with the cooling phase (cooling first) for 14 participants **(A)**. The corresponding average time-series data for the air temperature in the canopy **(B)** and the two subjective measures [i.e., sensation and comfort/uncomfortable ratings: **(C,D)** respectively] are shown separately for the heating first (solid line) and cooling first (dashed line) groups. Error bars indicate standard deviations.

Among the 28 participants whose data were analyzed, the fMRI session began with the heating phase for 14 participants and with the cooling phase for 14 participants (Figure 1A). The air temperature in the canopy increased and decreased almost linearly during the heating and cooling phases, respectively (Figure 1B). The thermal sensation rating (Figure 1C) generally changed in parallel with the inside-canopy temperature and tended to increase and decrease within each 10 min manipulation period. The thermal comfort rating (Figure 1D) was associated with the maximum discomfort at the hottest and coldest peak of the sensation rating with the highest degree reported during the transient periods between the peaks.

A tendency of and significant negative correlations were detected between the average comfort rating and the MTASQ score for motivational decline (F1) and the proactive response (F3), respectively; that is, participants with the higher scores on these two MTASQ dimensions perceived the thermal environment as more uncomfortable during the fMRI session (Table 3). No significant correlation was observed for the sensation rating during the fMRI task or the MTASQ score of the active behavior (F2) dimension.

**TABLE 3:** Correlation between the MTASQ scores and the comfort and sensation ratings.

	Comfort rating		Sensation (heat) rating	
	ρ	*p*	ρ	*p*
Motivational decline	-0.36	0.06^†^	-0.02	0.93
Active behavior	0.10	0.60	0.18	0.35
Proactive response	-0.39	0.04*	-0.20	0.32

Spearman’s correlation coefficients (ρ) between the MTASQ rating scores and the average thermal comfort and sensation (heat) ratings during the fMRI experiment. MTASQ: Multidimensional Thermal Adaption Style Questionnaire.

*p < 0.05, ^†^ < 0.10.

#### 3.2.2. First approach: Discomfort-related activation and the effect of the MTASQ score

**FIGURE 2:**
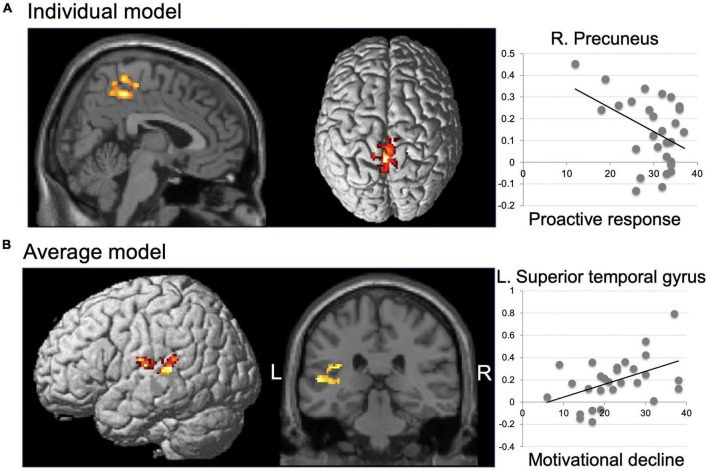
Discomfort-related activation and the effect of the MTASQ score (first approach). Left panels, significant activation in the individual model **(A)** and the average model **(B)** are superimposed on the standard SPM12 anatomical brain. Right panel, estimate (β) of discomfort-related activation (vertical axis) of each individual plotted against the significantly associated MTASQ score (horizontal axis); that is, the proactive response for the individual model **(A)** and motivational decline for the average model **(B)**; regression line is given for each plot. R, right; L, left.

Discomfort-related activation and the MTASQ effect at each peak activation are summarized in Table 4 for the first approach to the fMRI data. Discomfort-related activation, but not deactivation, was observed for the individual and the average models. Activation in the individual model was detected in the right precuneus and was higher for the low proactive response (F3) individuals (Figure 2A). In the average model, activation was observed in the left STG and was higher for individuals with high motivational decline (F1) (Figure 2B).

**TABLE 4:** Discomfort-related activation and the effect of the MTASQ score (first approach).

Brain region	Peak				Cluster		Another model (t)		Effect of MTASQ scores (t)				
	x	y	z	t	k	p			Motivational decline		Active behavior	Proactive response	
**Individual model**													
R. Precuneus	4	−38	48	4.99	657	0.001	3.20	*				−1.89	*
**Average model**													
L. STG	−54	−30	8	4.61	433	0.002	2.22	*	3.11	*			

The coordinates (x, y, z) and *t*-value of peak activation, and size and its *p*-value of the cluster are given for discomfort-related activation (voxel-wise one-sample *t*-test), separately for the individual and average models. At each peak voxel, the *t*-value is given for discomfort-related activation on another model (i.e., average and individual models for the peak obtained on individual and average models, respectively), as well as *t*-values for significant effects of MTASQ scores (regression analyses separately for three dimensions). L, left; R, right; STG, superior temporal gyrus. *p < 0.05, uncorrected.

#### 3.2.3. Second approach: Voxel-wise search of MTASQ effect on discomfort-related activation

**FIGURE 3:**
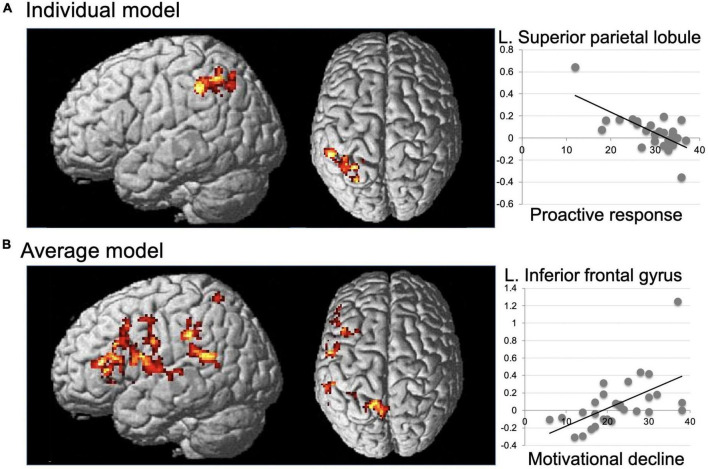
Significant effects of the MTASQ score on discomfort-related activation (second approach). Left panels, significant negative effect of the proactive response on individual-model-derived discomfort-related activation **(A)** and significant positive effect of motivational decline on average-model-derived activation **(B)** are superimposed on the standard anatomical brain. Other details are the same as for Figure 2.

The results of the voxel-wise regression analyses of each MTASQ score on discomfort-related activation are summarized in Table 5 for the second approach. For the individual model, a significant negative effect of proactive response (F3) was identified in a large cluster in the left lateral parietal cortex with peaks in the superior parietal lobule, angular gyrus, and supramarginal gyrus (Figure 3A). The observed negative effect in the superior parietal lobule was replicated in the average model at a liberal statistical threshold. A significant positive effect of motivational decline (F1) was identified in the left frontal cortex for the average model, with peaks in the inferior frontal gyrus and middle frontal gyrus, STG, and superior parietal lobule (Figure 3B). The observed positive effect in the middle frontal gyrus and STG was replicated in the average model at a liberal statistical threshold.

**TABLE 5:** Significant effects of the MTASQ score on discomfort-related activation (second approach).

MTASQ dimensions	Brain region	Peak				Cluster		Another model (t)	
		x	y	z	t	k	p		
**Individual model**									
Motivational decline	*n.s.*								
Active behavior	*n.s.*								
Proactive response	L. Superior parietal lobule	−30	−54	52	−5.34	506	0.001	−1.72	*
	L. Angular gyrus	−30	−66	42	−4.33				
	L. Supramarginal gyrus	−38	−46	42	−4.01				
**Average model**									
Motivational decline	L. Inferior frontal gyrus	−50	28	8	4.87	464	0.001		
	L. Middle frontal gyrus	−46	32	14	4.58			1.72	*
	L. Superior temporal gyrus	−50	−50	18	4.19	852	0.001	1.93	*
	L. Superior parietal lobule	−14	−56	56	4.58	257	0.029		
Active behavior	*n.s.*								
Proactive response	*n.s.*								

The results of the voxel-wise exploration of the significant effect of the MTASQ scores (i.e., regression analysis) on discomfort-related activation. At each peak voxel, the *t*-value for the effect of the same MTASQ score on discomfort-related activation in another model is given when significant. Other details are the same as for Table 4.

## 4. Discussion

In this study, we aimed at formulating major dimensions of the individual difference in responses and coping strategies to thermal environmental stress as well as understand the neural correlates of each factor. We first created a multidimensional inventory of individual difference in response and coping strategies to thermal environmental stress (i.e., MTASQ). We then examined how each factor was reflected in the neural response under environmental thermal stress using fMRI. We identified three factors for the MTASQ: motivational decline (F1), active behavior (F2), and the proactive response (F3). These factors were associated with different sets of apparently reasonable demographic, physiological, and psycho-behavioral variables, suggesting their construct validity. In addition, motivational decline factor (F1) score was positively associated with common neural response to thermal stress in the frontal and temporoparietal regions, implicated in emotion regulation, while proactive response factor (F3) score negatively with the neural responses related to subjective discomfort in the medial and lateral parietal cortices, implicated in problem-solving.

### 4.1. MTASQ factors

Motivational decline (F1) seems to reflect an inefficiency in the use of an adaptive emotion-focused coping strategy under environmental thermal stress. This factor was negatively associated with extraversion, agreeableness, conscientiousness, and subjective tolerance for a hot environment, and positively associated with neuroticism, subjective intolerance to hot or cold temperatures, and minimal tolerable high temperature. The combination of high neuroticism combined with low extraversion, agreeableness, and conscientiousness constitutes the most maladaptive personality type among participants who report more severe childhood maltreatment, such as emotional neglect or emotional abuse (Spinhoven et al., 2016) and followed a review study showing that the predominance of these personality traits predisposes to maladaptive emotion-focused coping (Carver and Connor-Smith, 2010). In particular, neuroticism is consistently positively associated with passive and ineffective coping mechanisms and negatively associated with the acceptance of the reality of what has happened or learning something useful from an experience (Watson and Hubbard, 1996). Neurotic individuals, who are stress-prone, react to stress more intensely, leading to emotional exhaustion (Hudek-Knežević et al., 2006), which may explain the positive association between the decline in motivation and subjective intolerance to hot or cold temperature or minimal tolerable high temperature in our study.

Active behavior (F2) was difficult to attribute to either an emotion-focused or problem-focused coping strategy. It appeared that individuals with this trait perceive high or low temperatures within the daily life range not as stress but as an opportunity for exciting or enjoyable activities. A sauna or taking a cold shower is recognized as a pleasurable activity with an additional health benefit (Heinonen and Laukkanen, 2018; Schmid, 2018). This view is consistent with the positive association between this factor score and subjective tolerance to hot and cold temperatures. Temperature tolerance may be related to relatively high adaptability of humans to hot and cold environments (Heinonen and Laukkanen, 2018). It is also understood that such tolerance is positively associated with many survival-relevant characteristics (i.e., factors of the “power to live”).

The proactive response (F3) appeared to reflect individual efficiency in taking a problem-focused coping strategy related to thermal stress. This factor was positively associated with female sex and prosocial personality traits (i.e., altruism and agreeableness), consistent with a previously reported association between problem-focused coping and prosocial behaviors in females (Khamis, 2018); other associated power-to-live factors, such as problem-solving, etiquette, and self-transcendence, have been shown to enhance pro-social helping behavior (Sugiura et al., 2020). The remaining associated power-to-live factors, such as stubbornness, emotion regulation, and active wellbeing, are associated with better physical and mental health (Sugiura et al., 2015), consistent with the previously reported associations between problem-focused coping and the maintenance of positive affect (Folkman and Moskowitz, 2000), wellbeing (Mayordomo-Rodríguez et al., 2015), and solving problems that elicit negative emotions (Sun et al., 2021).

### 4.2. fMRI data

#### 4.2.1. Behavioral data

The negative correlations between the average comfort rating and the scores of the two MTASQ factors, including motivational decline (F1) and proactive response (F3), were reasonable given their likely association with stress coping. The correlation with motivational decline is consistent with the previously demonstrated relationship between emotion-focused coping tendency and sensitivity to discomfort (Amen, 2008; Schoenmakers et al., 2015). The correlation with proactive response was consistent with our hypothesis that problem-focused coping is triggered by subjectively perceived thermal discomfort.

#### 4.2.2. Motivational decline

The neural finding for motivational decline agrees with the view that this factor reflects an individual tendency for the use of an emotion-focused coping strategy. First, the factor score was primarily correlated with discomfort-related activation in the average model. It is congruent with our hypothesis that emotion-focused coping taps into the early stage, first-level valuation before second-level valuation or emotional responses develop. Second, the correlation was identified in the frontal, parietal, and temporal cortices previously implicated in emotion regulation (Ochsner et al., 2012; Picó-Pérez et al., 2017). Specifically, the VLPFC plays a role in emotional inhibition; the DLPFC and superior parietal lobule play roles in attentional control, and the STG is involved in modulating the generation of emotions through perceptual and semantic processing (Ochsner et al., 2012).

Our findings suggest that motivational decline reflects inefficiency in the use of the emotion-focused coping strategy. These areas were more activated in individuals with a large decline in motivation, suggesting that activation reflects more difficulties in regulating the emotional response to environmental thermal stress. The finding is consistent with previous observations that show less activation in these regions in adaptive individuals during the task under potential emotional stress (Preis et al., 2015; Kober et al., 2019; Messina et al., 2021).

#### 4.2.3. Proactive response

The neural finding for proactive response agrees with the view that this factor reflects the tendency to follow a problem-focused coping strategy in an uncomfortable thermal environment. First, the factor score was correlated with discomfort-related activation in the individual model. It is congruent with our hypothesis that problem-focused coping is responsive to the subjective perception of thermal discomfort related to late-stage, second-level valuation, or an emotional response. Second, a correlation was identified in the medial and lateral parietal cortices, which has been implicated in a wide range of creative problem-solving (Bartley et al., 2018) and future thinking (Stawarczyk and D’Argembeau, 2015; Schacter et al., 2017). These skills are likely to be involved in the initial planning process for problem-focused coping.

Our findings suggest that a proactive response reflects the efficiency of the problem-focused coping process. Minimal activation of the medial and lateral parietal cortices in individuals with a high factor score is likely to be related to their efficiency in such a planning process. They may spend few cognitive resources thinking of appropriate and practical measures to address climate-related problems in daily life, such as collecting necessary information (i.e., weather forecast) or preparing for a relevant health concern (e.g., dehydration, coldness, sunburn, and dryness). The suggested association between adaptability and low activation follows the previous observation that high problem-solving performance is associated with low activation in these areas (Miura et al., 2020; Oba et al., 2020).

### 4.3. Theoretical and practical implications

Our results thus demonstrated that individual differences in the way people respond to and cope with thermal environmental stress can be summarized into three dimensions in part congruent with the stress-coping model by Lazarus and Folkman (Folkman and Lazarus, 1980, 1985); motivational decline (F1) and proactive response (F3) were related to emotion-focused and problem-focused strategies, respectively. The correspondence was supported by the related psycho-behavioral variables and neural correlates under the environmental thermal stress in this study. The results may also have academic significance in demonstrating the generalizability of Lazarus and Folkman’s model for environmental thermal stress. In addition, the three dimensions results will expand the frontiers of meteorological human science in various directions. Previous studies in this field have explored the factors affecting environmental thermal stress primarily on population bases. They included environmental factors (Shi et al., 2016; Gao et al., 2018), physiological and demographic factors (Foster et al., 2020), and technical interventions (Coudevylle et al., 2019). Although the necessity of including individual psycho-behavioral factors has been suggested (Nikolopoulou et al., 2001; Coudevylle et al., 2019), a relevant theoretical framework has not been available. The current three-dimensional framework may fill this gap.

The current three-dimensional framework may have practical implications in understanding how people respond and prefer to cope with thermal environmental stress, which need is more pressing than ever in the increasing prevalence of temperature change on earth and movement of people across intercontinental borders. For example, the answers to these questions will help develop optimization technology for the thermal environment on an individual basis. We may identify an air-conditioning set-point to balance subjective discomfort and energy efficiency based on individual levels of motivational decline (F1). In the design of living spaces and cities, a more comfortable thermal environment could be achieved by adjusting the behavior of users based on individual levels of proactive responses (F3). Finally, further research on the active behavior (F2) dimension from an anthropological perspective may shed new light on this topic.

### 4.4. Strengths and limitations

The strengths of study are that we first identified three dimensions in how people react to and cope with thermal environmental stress, of which two complied with an influential two-dimensional framework for stress coping developed by Lazarus and Folkman (Folkman and Lazarus, 1980, 1985). There were also several limitations. First, one major limitation of the present study is the sample size for the fMRI experiment. We only included 28 participants in the final fMRI analysis. However, considering the relatively long scanning time and duration of each condition, it may be inappropriate to simply compare the statistical characteristics of the current study with simulation data from studies with a larger number of participants. Second, the limited temperature range. More severe thermal stress could have provided different results. Third, the representativeness of our participants was limited. As we only recruited Japanese demographic profiles, our results may not cover the psycho-behavioral characteristics that exist in other populations with other geographic, physiological, or socio-cultural profiles.

## 5. Conclusion

We identified three dimensions among the responses to environmental thermal stress, as formulated in the MTASQ. Motivational decline (F1) reflected individual inefficiency in the use of an adaptive emotion-focused coping strategy under thermal stress. Active behavior (F2) reflected the tendency to perceive high or low thermal temperature not as stress but as an opportunity for some exciting or enjoyable activity. Proactive responses (F3) reflected individual efficiency in taking a problem-focused coping strategy related to environmental thermal stress. Our neural findings on the correlation of these factor scores and discomfort-related activation provide further support for the association between motivational decline (F1) and inefficient emotion-focused coping in terms of its relevance to high early stage (i.e., average model) activation in the frontal and temporoparietal regions, which has been implicated in emotion regulation. Although the active behavior (F2) was associated with many survival-relevant characteristics (i.e., “power to live” factors), we failed to identify its neural correlates in terms of the response to environmental thermal stress. The expression of this trait dimension may be triggered by neural dynamics other than stress perception. The association between the proactive response (F3) and efficient problem-focused coping was also supported by its relevance to low late-stage (i.e., individual model) activation in the medial and lateral parietal cortices, which has been implicated in creative problem-solving and future thinking. Thus, within the context of environmental thermal stress and as the theoretical implication, we have shown that motivational decline and proactive response factors were conformed to an influential two-dimensional framework of stress coping by Lazarus and Folkman (Folkman and Lazarus, 1980, 1985). An important practical implication is that the current three-dimensional model may expand the frontiers of meteorological human science in both basic and application domains.

## Data availability statement

The raw data supporting the conclusions of this article are not publicly available due to the risk of identifying the participants from the reconstructed images. However, the data are available from the corresponding author upon a reasonable request.

## Ethics statement

The studies involving human participants were reviewed and approved by the Institutional Review Board of the Tohoku University, Graduate School of Medicine, and the Nissan Motor Ethics Committee. Written informed consent to participate in this study was provided by the participants’ legal guardian/next of kin.

## Author contributions

KHdSK and MS conceived the experiments, analyzed the data, and drafted the manuscript. KHdSK, KH, YH, AK, and MS collected the data. KHdSK, KH, YH, AK, RK, and MS critically reviewed and approved the final version of the manuscript. All authors contributed to the article and approved the submitted version.

## References

[B1] Agbaria Q. Mokh A. A. (2022). Coping with stress during the coronavirus outbreak: The contribution of big five personality traits and social support. *Int. J. Ment. Health Addict.* 20 1854–1872. 10.1007/s11469-021-00486-2 33500687 PMC7819145

[B2] Aldao A. Nolen-Hoeksema S. (2012). When are adaptive strategies most predictive of psychopathology? *J. Abnorm. Psychol.* 121 276–281. 21553934 10.1037/a0023598

[B3] Aldao A. Nolen-Hoeksema S. Schweizer S. (2010). Emotion-regulation strategies across psychopathology: A meta-analytic review. *Clin. Psychol. Rev.* 30 217–237. 20015584 10.1016/j.cpr.2009.11.004

[B4] Amen A. (2008). *Monitoring the mind: The relationship between individual differences in cognitive control and emotion regulation.* Doctoral thesis. Haverford, PA: Haverford College.

[B5] Bartley J. E. Boeving E. R. Riedel M. C. Bottenhorn K. L. Salo T. Eickhoff S. B. (2018). Meta-analytic evidence for a core problem solving network across multiple representational domains. *Neurosci. Biobehav. Rev.* 92 318–337. 10.1016/j.neubiorev.2018.06.009 29944961 PMC6425494

[B6] Carver C. S. Connor-Smith J. (2010). Personality and coping. *Annu. Rev. Psychol.* 61 679–704. 19572784 10.1146/annurev.psych.093008.100352

[B7] Chen Y. Peng Y. Xu H. O’Brien W. H. (2018). Age differences in stress and coping: Problem-focused strategies mediate the relationship between age and positive affect. *Int. J. Aging Hum. Dev.* 86 347–363. 10.1177/0091415017720890 28789561

[B8] Cohen J. (1992). A power primer. *Psychol. Bull.* 112 155–159. 19565683 10.1037//0033-2909.112.1.155

[B9] Comrey A. L. (1988). Factor-analytic methods of scale development in personality and clinical psychology. *J. Consult. Clin. Psychol.* 56 754–761. 3057010 10.1037//0022-006x.56.5.754

[B10] Coudevylle G. R. Sinnapah S. Robin N. Collado A. Hue O. (2019). Conventional and alternative strategies to cope with the subtropical climate of Tokyo 2020: Impacts on psychological factors of performance. *Front. Psychol.* 10:1279. 10.3389/fpsyg.2019.01279 31214085 PMC6558207

[B11] Cui H. Jeong H. Okamoto K. Takahashi D. Kawashima R. Sugiura M. (2022). Neural correlates of Japanese honorific agreement processing mediated by socio-pragmatic factors: An fMRI study. *J. Neurolinguistics* 62:101041.

[B12] Diponegoro A. M. Santoso A. M. Nurjannah E. S. Diastu N. R. Ali K. Fidyawaty Y. (2020). Problem focused coping methods used by students during Covid-19. *KnE Soc. Sci.* 4 109–120.

[B13] Eickhoff S. B. Stephan K. E. Mohlberg H. Grefkes C. Fink G. R. Amunts K. (2005). A new SPM toolbox for combining probabilistic cytoarchitectonic maps and functional imaging data. *Neuroimage* 25 1325–1335. 10.1016/j.neuroimage.2004.12.034 15850749

[B14] Ellis P. D. (2010). *The essential guide to effect sizes: Statistical power, meta-analysis, and the interpretation of research results.* Cambridge: Cambridge University Press.

[B15] Folkman S. Lazarus R. S. (1980). An analysis of coping in a middle-aged community sample. *J. Health Soc. Behav.* 21 219–239. 7410799

[B16] Folkman S. Lazarus R. S. (1985). If it changes it must be a process: Study of emotion and coping during three stages of a college examination. *J. Pers. Soc. Psychol.* 48 150–170. 2980281 10.1037//0022-3514.48.1.150

[B17] Folkman S. Moskowitz J. T. (2000). Positive affect and the other side of coping. *Am. Psychol.* 55 647–654. 10892207 10.1037//0003-066x.55.6.647

[B18] Foster J. Hodder S. G. Lloyd A. B. Havenith G. (2020). Individual responses to heat stress: Implications for hyperthermia and physical work capacity. *Front. Physiol.* 11:541483. 10.3389/fphys.2020.541483 33013476 PMC7516259

[B19] Friston K. Frith C. Liddle P. Dolan R. J. Lammertsma A. Frackowiak R. (1990). The relationship between global and local changes in PET scans. *J. Cereb. Blood Flow Metab.* 10 458–466. 2347879 10.1038/jcbfm.1990.88

[B20] Friston K. J. Frith C. Liddle P. Frackowiak R. (1991). Comparing functional (PET) images: The assessment of significant change. *J. Cereb. Blood Flow Metab.* 11 690–699. 2050758 10.1038/jcbfm.1991.122

[B21] Fukusaka M. Matsubara N. (2014). Actual conditions of residents’ cooling behaviors related to visual and auditory sensation and estimation of their effect on energy saving in Japan. *J. Hum. Environ. Syst.* 17 13–24.

[B22] Gao C. Kuklane K. Östergren P. O. Kjellstrom T. (2018). Occupational heat stress assessment and protective strategies in the context of climate change. *Int. J. Biometeorol.* 62 359–371. 10.1007/s00484-017-1352-y 28444505 PMC5854720

[B23] Gosling S. D. Rentfrow P. J. Swann W. B. Jr. (2003). A very brief measure of the big-five personality domains. *J. Res. Pers.* 37 504–528.

[B24] Gross J. J. (1998a). Antecedent-and response-focused emotion regulation: Divergent consequences for experience, expression, and physiology. *J. Pers. Soc. Psychol.* 74 224–237. 10.1037//0022-3514.74.1.224 9457784

[B25] Gross J. J. (1998b). The emerging field of emotion regulation: An integrative review. *Rev. Gen. Psychol.* 2 271–299.

[B26] Hanna J. M. Brown D. E. (1983). Human heat tolerance: An anthropological perspective. *Annu. Rev. Anthropol.* 12 259–284.

[B27] Heinonen I. Laukkanen J. A. (2018). Effects of heat and cold on health, with special reference to finnish sauna bathing. *Am. J. Physiol. Regul. Integr. Comp. Physiol.* 314 R629–R638. 10.1152/ajpregu.00115.2017 29351426

[B28] Ho S. Sun D. Ting K. Chan C. Lee T. (2015). Mindfulness trait predicts neurophysiological reactivity associated with negativity bias: An ERP study. *Evid. Based Complement. Alternat. Med.* 2015:212368. 10.1155/2015/212368 26124852 PMC4466385

[B29] Hudek-Knežević J. Krapić N. Kardum I. (2006). Burnout in dispositional context: The role of personality traits, social support and coping styles. *Rev. Psychol.* 13 65–73.

[B30] Huynen M.-M. Martens P. Schram D. Weijenberg M. P. Kunst A. E. (2001). The impact of heat waves and cold spells on mortality rates in the Dutch population. *Environ. Health Perspect.* 109 463–470. 10.1289/ehp.01109463 11401757 PMC1240305

[B31] Ishibashi R. Nouchi R. Honda A. Abe T. Sugiura M. (2019). A concise psychometric tool to measure personal characteristics for surviving natural disasters: Development of a 16-item power to live questionnaire. *Geosciences* 9:366.

[B32] Jendritzky G. Tinz B. (2009). The thermal environment of the human being on the global scale. *Glob. Health Action* 2:2005. 10.3402/gha.v2i0.2005 PMC2799257 20052427

[B33] Kageyama T. dos Santos Kawata K. H. Kawashima R. Sugiura M. (2019). Performance and material-dependent holistic representation of unconscious thought: A functional magnetic resonance imaging study. *Front. Hum. Neurosci.* 13:418. 10.3389/fnhum.2019.00418 31866843 PMC6908964

[B34] Khamis V. (2018). How can gender affect psychopathology in Lebanese school-age children? *Psychol. Sch.* 55 404–418.

[B35] Kober H. Buhle J. Weber J. Ochsner K. N. Wager T. D. (2019). Let it be: Mindful acceptance down-regulates pain and negative emotion. *Soc. Cogn. Affect. Neurosci.* 14 1147–1158. 10.1093/scan/nsz104 31989171 PMC7057281

[B36] Koenigsberg H. W. Siever L. J. Lee H. Pizzarello S. New A. S. Goodman M. (2009). Neural correlates of emotion processing in borderline personality disorder. *Psychiatry Res.* 172 192–199. 19394205 10.1016/j.pscychresns.2008.07.010 PMC4153735

[B37] Laurent J. G. C. Williams A. Oulhote Y. Zanobetti A. Allen J. G. Spengler J. D. (2018). Reduced cognitive function during a heat wave among residents of non-air-conditioned buildings: An observational study of young adults in the summer of 2016. *PLoS Med.* 15:e1002605. 10.1371/journal.pmed.1002605 29990359 PMC6039003

[B38] Lazarus R. S. (1991). *Emotion and Adaptation.* Oxford: Oxford University Press.

[B39] Li G. Liu C. He Y. (2021). The effect of thermal discomfort on human well-being, psychological response and performance. *Sci. Technol. Built Environ.* 27 960–970.

[B40] Mann H. B. Whitney D. R. (1947). On a test of whether one of two random variables is stochastically larger than the other. *Ann. Math. Stat.* 18 50–60.

[B41] Matsuzaki Y. Ishibashi R. Yasuda M. Tanabe A. Honda A. Abe T. (2022). Does the eight-factor “power to live” in disaster exist since childhood? *Front. Public Health* 10:1022939. 10.3389/fpubh.2022.1022939 36579065 PMC9791042

[B42] Mayordomo-Rodríguez T. Meléndez-Moral J. C. Viguer-Segui P. Sales-Galán A. (2015). Coping strategies as predictors of well-being in youth adult. *Soc. Indic. Res.* 122 479–489.

[B43] Messina I. Grecucci A. Viviani R. (2021). Neurobiological models of emotion regulation: A meta-analysis of neuroimaging studies of acceptance as an emotion regulation strategy. *Soc. Cogn. Affect. Neurosci.* 16 257–267. 10.1093/scan/nsab007 33475715 PMC7943364

[B44] Miura N Yoshii K Takahashi M Sugiura M Kawashima R. (2020). Functional MRI on the ability to handle unexpected events in complex socio-technological systems: Task performance and problem-solving characteristics are associated with low activity of the brain involved in problem solving. *Trans. Hum. Interface Soc.* 22 43–54. 10.11184/his.22.1_43

[B45] Nikolopoulou M. Baker N. Steemers K. (2001). Thermal comfort in outdoor urban spaces: Understanding the human parameter. *Sol. Energy* 70 227–235. 10.1007/s00484-021-02136-7 33929628

[B46] Oba K. Sugiura M. Hanawa S. Suzuki M. Jeong H. Kotozaki Y. (2020). Differential roles of amygdala and posterior superior temporal sulcus in social scene understanding. *Soc. Neurosci.* 15 516–529. 10.1080/17470919.2020.1793811 32692950

[B47] Ochsner K. N. Silvers J. A. Buhle J. T. (2012). Functional imaging studies of emotion regulation: A synthetic review and evolving model of the cognitive control of emotion. *Ann. N. Y. Acad. Sci.* 1251 E1–E24. 10.1111/j.1749-6632.2012.06751.x 23025352 PMC4133790

[B48] Oi H. Hashimoto T. Nozawa T. Kanno A. Kawata N. Hirano K. (2017). Neural correlates of ambient thermal sensation: An fMRI study. *Sci. Rep.* 7:11279. 10.1038/s41598-017-11802-z 28900235 PMC5595885

[B49] Öner S. (2018). Neural substrates of cognitive emotion regulation: A brief review. *Psychiatry Clin. Psychopharmacol.* 28 91–96. 10.1016/0306-4530(95)00042-9 8774063

[B50] Oshio A. Shingo A. Cutrone P. (2012). Development, reliability, and validity of the Japanese version of ten item personality inventory (TIPI-J). *Jpn. J. Pers.* 21 40–52.

[B51] Picó-Pérez M. Radua J. Steward T. Menchón J. M. Soriano-Mas C. (2017). Emotion regulation in mood and anxiety disorders: A meta-analysis of fMRI cognitive reappraisal studies. *Prog. Neuropsychopharmacol. Biol. Psychiatry* 79 96–104. 10.1016/j.pnpbp.2017.06.001 28579400

[B52] Preis M. A. Kröner-Herwig B. Schmidt-Samoa C. Dechent P. Barke A. (2015). Neural correlates of empathy with pain show habituation effects. An fMRI study. *PLoS One* 10:e0137056. 10.1371/journal.pone.0137056 26317858 PMC4552664

[B53] Salomons T. V. Johnstone T. Backonja M.-M. Shackman A. J. Davidson R. J. (2007). Individual differences in the effects of perceived controllability on pain perception: Critical role of the prefrontal cortex. *J. Cogn. Neurosci.* 19 993–1003. 10.1162/jocn.2007.19.6.993 17536969

[B54] Sato S. Ishibashi R. Sugiura M. (2021). Two major elements of life recovery after a disaster: Their impacts dependent on housing damage and the contributions of psycho-behavioral factors. *J. Disaster Res.* 16 1107–1120.

[B55] Schacter D. L. Benoit R. G. Szpunar K. K. (2017). Episodic future thinking: Mechanisms and functions. *Curr. Opin. Behav. Sci.* 17 41–50. 29130061 10.1016/j.cobeha.2017.06.002 PMC5675579

[B56] Schmid J.-P. (2018). Some like it hot: Cardiovascular health benefits of Finnish sauna. *Eur. J. Prev. Cardiol.* 25 127–129. 10.1177/2047487317742501 29160085

[B57] Schoenmakers E. C. van Tilburg T. G. Fokkema T. (2015). Problem-focused and emotion-focused coping options and loneliness: How are they related? *Eur. J. Ageing* 12 153–161. 10.1007/s10433-015-0336-1 28804352 PMC5549139

[B58] Shi L. Liu P. Wang Y. Zanobetti A. Kosheleva A. Koutrakis P. (2016). Chronic effects of temperature on mortality in the Southeastern USA using satellite-based exposure metrics. *Sci. Rep.* 6:30161. 10.1038/srep30161 27436237 PMC4951799

[B59] Shin H. Park Y. M. Ying J. Y. Kim B. Noh H. Lee S. M. (2014). Relationships between coping strategies and burnout symptoms: A meta-analytic approach. *Prof. Psychol. Res. Pract.* 45:44.

[B60] Spearman C. (1961). “The proof and measurement of association between two things,” in *Studies in Individual Differences: The Search for Intelligence*, eds Jenkins J. J. Paterson D. G. (New York, NY: Appleton-Century-Crofts), 45–58.

[B61] Spinhoven P. Elzinga B. M. Van Hemert A. M. de Rooij M. Penninx B. W. (2016). Childhood maltreatment, maladaptive personality types and level and course of psychological distress: A six-year longitudinal study. *J. Affect. Disord.* 191 100–108. 10.1016/j.jad.2015.11.036 26655119

[B62] Stawarczyk D. D’Argembeau A. (2015). Neural correlates of personal goal processing during episodic future thinking and mind-wandering: An ALE meta-analysis. *Hum. Brain Mapp.* 36 2928–2947. 10.1002/hbm.22818 25931002 PMC6869624

[B63] Sugiura M. (2022). Adaptability, supernaturalness, and the neurocognitive basis of the self-transcendence trait: Toward an integrated framework through disaster psychology and a self-agency model. *Front. Behav. Neurosci.* 16:943809. 10.3389/fnbeh.2022.943809 36062259 PMC9435587

[B64] Sugiura M. Ishibashi R. Abe T. Nouchi R. Honda A. Sato S. (2021). Self-help and mutual assistance in the aftermath of a tsunami: How individual factors contribute to resolving difficulties. *PLoS One* 16:e0258325. 10.1371/journal.pone.0258325 34618878 PMC8496872

[B65] Sugiura M. Nouchi R. Honda A. Sato S. Abe T. Imamura F. (2020). Survival-oriented personality factors are associated with various types of social support in an emergency disaster situation. *PLoS One* 15:e0228875. 10.1371/journal.pone.0228875 32050260 PMC7015700

[B66] Sugiura M. Sato S. Nouchi R. Honda A. Abe T. Muramoto T. (2015). Eight personal characteristics associated with the power to live with disasters as indicated by survivors of the 2011 Great East Japan Earthquake disaster. *PLoS One* 10:e0130349. 10.1371/journal.pone.0130349 26132753 PMC4488507

[B67] Sugiura M. Sato S. Nouchi R. Honda A. Ishibashi R. Abe T. (2019). Psychological processes and personality factors for an appropriate tsunami evacuation. *Geosciences* 9:326.

[B68] Sun Y. Li Y. Wang Y. Li F. (2021). Understanding the emotion coping strategies during public emergencies - from the perspective of psychological distance. *Front. Psychol.* 12:699180. 10.3389/fpsyg.2021.699180 34803796 PMC8603827

[B69] Tham K. W. (2004). Effects of temperature and outdoor air supply rate on the performance of call center operators in the tropics. *Indoor Air* 14 119–125. 15330779 10.1111/j.1600-0668.2004.00280.x

[B70] Tochihara Y. Wakabayashi H. Lee J.-Y. Wijayanto T. Hashiguchi N. Saat M. (2022). How humans adapt to hot climates learned from the recent research on tropical indigenes. *J. Physiol. Anthropol.* 41:27. 10.1186/s40101-022-00302-3 35836266 PMC9281079

[B71] Van Zomeren M. Spears R. Leach C. W. (2010). Experimental evidence for a dual pathway model analysis of coping with the climate crisis. *J. Environ. Psychol.* 30 339–346.

[B72] Vargha A. Delaney H. D. (2000). A critique and improvement of the CL common language effect size statistics of McGraw and Wong. *J. Educ. Behav. Stat.* 25 101–132.

[B73] Wang Z. de Dear R. Luo M. Lin B. He Y. Ghahramani A. (2018). Individual difference in thermal comfort: A literature review. *Build. Environ.* 138 181–193.

[B74] Watson D. Hubbard B. (1996). Adaptational style and dispositional structure: Coping in the context of the five-factor model. *J. Pers.* 64 737–774.

[B75] Wolters G. Stapert S. Brands I. Van Heugten C. (2010). Coping styles in relation to cognitive rehabilitation and quality of life after brain injury. *Neuropsychol. Rehabil.* 20 587–600. 20446171 10.1080/09602011003683836

[B76] Zhang F. de Dear R. Hancock P. (2019). Effects of moderate thermal environments on cognitive performance: A multidisciplinary review. *Appl. Energy* 236 760–777. 10.1186/s12913-016-1423-5 27409075 PMC4943498

