# Supplementary material for: Motivational decline and proactive response under thermal environmental stress are related to emotion- and problem-focused coping, respectively: Questionnaire construction and fMRI study
Source: Front Behav Neurosci. 2023 Apr 12;17:1143450. doi: 10.3389/fnbeh.2023.1143450 (PMC10130452; doi:10.3389/fnbeh.2023.1143450)
Supplement: Supplementary file 1 [file Table_1.DOCX]

Supplementary Material

Motivational decline and proactive response under thermal environmental stress are
related to emotion- and problem-focused coping, respectively: Questionnaire construction and fMRI study

Kelssy Hitomi dos Santos Kawata, Kanan Hirano, Yumi Hamamoto, Hajime Oi, Akitake Kanno, Ryuta Kawashima, Motoaki Sugiura*

*** Correspondence:** Motoaki Sugiura: motoaki.sugiura.d6@tohoku.ac.jp

**Supplementary Table 1.** Responders of the two surveys for constructing the MTASQ.

|  | Qualitative survey | | Quantitative survey | |
| --- | --- | --- | --- | --- |
|  | Male | Female | Male | Female |
| 20s | 22 | 20 | 114 | 140 |
| 30s | 21 | 20 | 117 | 144 |
| 40s | 20 | 20 | 124 | 138 |
| 50s | 20 | 20 | 128 | 137 |
| 60s | 20 | 20 | 140 | 145 |
| total | 203 | | 1327 | |

We intended to have 20 and 120 respondents for each class after data pre-screening (excluding straight-line responders)in the qualitative and quantitative surveys, respectively.
